# Bibliographical Notices

**Published:** 1853-06

**Authors:** 


### BIBLIOGRAPHICAL NOTICES.

---

*Principles of Organic and Physiological Chemistry.* By CARL LÖWIG, M. D.; Ph. D., Ordinary Professor of Chemistry at the University of Zurich, &c. Translated by Daniel Breed, M. D., of the U. S. Patent Office. Philada. A. Hart: 1853. pp. 481, octavo.

We confess to a feeling of satisfaction in looking over this book, arising chiefly from gratification from having so rich a vein of German chemical literature opened to our free access, yet not a little, also, from the American origin of the translation. Dr. Breed, whilst a student with Löwig, conceived the idea of translating this work of his preceptor, and executed a large portion of it whilst enjoying personal intercourse with the author. The exceeding fullness of the terminology and the peculiar views of the author, as exhibited in his endeavor to substitute rational for empirical formulæ, has rendered the task one of great difficulty; yet we believe it has been executed in a manner to do justice to Dr. Löwig, and great credit to the translator. Further, the book itself is well "gotten up" by the publisher, and is quite as free from errors of the press as such complex typography is usually found to be. Let us now look at the volume as Dr. Breed has unfolded it to us, and see if within the space allowed to this notice we can convey an idea of its character.

Dr. Löwig is the author of "Chemie der Organischen Verbindungen," (an octavo of 3000 pages,) which is now passing through its third edition. The present work may be looked upon as an introduction to that great work, and is intended to explain more fully the principles adopted in it, especially those which, originated with the author. For this reason many of the details common in works on organic chemistry, as the names of discoverers, manufacturing processes, etc., are omitted. The "Principles" is in two parts; the first is devoted to explaining the constitution and formation of organic compounds in general, and the constitution of organic radicals in particular, and is an essay on the philosophy of organic chemistry, of the highest order. The immense accumulation of observations on organic substances and products renders it absolutely necessary to adopt wider and more simple generalizations than have yet been offered, by which the phenomena of organic combinations may be classed in a more harmonious relation, and the laws which govern and control their intricate affinities developed, so as to impress the beautiful simplicity of a natural arrangement on the vast heterogeneous mass, and marshal it into order. The work of our author is an attempt in this direction. He is a thorough advocate of the Theory of Radicals; not as originally broached by Berzelius, but much modified, so as to harmonize many facts that are prominent in the views of Dumas, Laurent, and Gerhardt, as exhibited in the doctrine of Substitution, and the Theories of Types and Nuclei. There are two ways of viewing the formula or symbolical representation of the composition of an organic compound. In the first, the number of atoms of each element is simply indicated; thus $C_8\ H_8\ O_4$ represents the elementary constituents of acetic ether, and is its *empirical* formula, or just what would be obtained by an analysis, supposing it presented to a chemist for the first time, and before any attempt at discovering its nature was made. In the second, the atoms are grouped so as to exhibit the proximate constitution of the substance, thus:—$C_4\ H_5\ O+C_4\ H_3\ O_3$ indicates that the substance is a compound of ether and acetic acid; whilst $C_4\ H_5,\ O+C_4\ H_3,\ O_3$ explains further that ether is the oxide of the radical ethyl, and that acetic acid is the oxide of the radical acetyl, and is its *rational* formula.

A radical is that part of a compound which preserves its identity when the other part or parts are removed and replaced by other elements or substances: For instance, nitrogen, in the five compounds which it forms with 1, 2, 3, 4 and 5 equivalents of oxygen, is a *simple radical*, whilst the substance $C_4\ H_5$ in ether is called a *compound* (or organic) *radical*, because the atom of oxygen, which by union constitutes it ether, may be replaced by chlorine, bromine or sulphur, without destroying the *essential* affinity of its atoms. Such radicals our author calls *primary* organic radicals. Now, a second series of radicals are obtained from these by the substitution, partially or wholly, of other elements for the hydrogen. Thus—acetyl$=C_4\ H_3$ is under favorable circumstances acted on by chlorine, which displaces its hydrogen, forming three equivalents of hydro-chloric acid, and replaces the hydrogen with equivalent atoms of chlorine, so as to produce $C_4\ Cl_3 =$ chloracetyl. Now, in this reaction the *specific atomic affinity* of the acetyl remains undisturbed, whilst the matter itself has been changed, and the resulting chloracetyl acts the part of a radical, forming chlor-acetic acid with oxygen, and a chloride with chlorine, just as acetyl does, yet possessing different properties. It is therefore called by our author a *derived* radical. In the instance mentioned the substitution has been complete; in other cases it is only partial, as where valeryl $=$ $C_{10}\ H_9$ becomes by partial displacement of hydrogen atoms $C_{10} \begin{matrix} H_7 \\ Cl_2 \end{matrix}$ and further, $C_{10} \begin{matrix} H_5 \\ Cl_4 \end{matrix}$. Owing to this *law of substitution* the derivative compounds are exceedingly numerous, yet such a mutual relation exists among the substances traceable to a common primary radical, that, as an entire group, they constitute a *natural family*, just as the botanical affinities of plants constitute a family in the natural system of Jussieu.

Radicals may be considered as the elements in inorganic compounds, and may be called compound atoms acting as a whole. It frequently happens that two or even more of these compound atoms unite to form a radical, in which case the combining capacity of the compound is generally determined by but one of them. Such are called *paired radicals* and the indifferent body the *pairling*. Thus, $C_6\ H_3 = (C_4\ H_2) \frown C_2H$, in which $C_4\ H_2$ is the passive molecule and $C_2H$ the active one, the former being enclosed in brackets and connected with the other by a hyphen. If, as occasionally happens, two active radicals unite, a *double radical* results, as in succinic acid, wherein $C_8\ H_4\ O_6 = (C_6\ H_3)\ O_3 \frown (C_2H)\ O_3$ the resulting acid being bi-basic and is a true double acid.

Radicals are sometimes *isomeric*, that is, have the same elementary composition, yet, in combining with the same substances, yield compounds varying in properties. Thus, $C_{14}\ H_5$ represents the composition of the radicals of benzoic and salicylous acids, yet, while in the first the formula represents a simple radical, the second is a paired radical consisting of $(C_4\ H_2) \frown C_{10}\ H_3$.

Other radicals are *polymeric* when their relative proportion of atoms is the same, but their absolute proportion different, as in the hydro-carbon volatile oils; and in yet other compounds, though the absolute and relative number of the atoms is the same, they have a different proximate arrangement and are called *metameric.* · Thus, hydrated acetic acid and formate of methyl are both composed of $C_4\ H_4\ O_4$, yet the formula of the former is $HO, (C_4\ H_3)\ O_3$, whilst that of the latter is $(C_2\ H_3)\ O, (C_2H)\ O_3$.

The Chapter of Dr. Löwig on the formation of organic compounds is replete with original observations and possessed of unusual interest. It is the highest aim of the Organic Chemist to unfold the ultimate constitution of bodies, and then trace back their constituents to their simplest forms. The various affinities, each in its sphere of action among definite atoms or groups of atoms, which are the uniting influence that impresses proximate character on the endless variety of chemical products, constitute a study of the deepest interest. The primitive affinities are the strongest and most permanent, and give rise to organic substances with but few atoms, whilst under the various degrees of secondary affinity the more complex and less permanent compounds result. The formation of organic compounds results,

1st, From the decomposition of inorganic substances, generally under the influence of the vitality of plants with the co-operation of light, but in some instances by artificial means ; and,

2d, By the decomposition of other organic compounds by means of manifold influences.

The formation of organic compounds in the vegetable organism from carbonic acid and ammonia is the part of vegetable physiology most interesting to the chemist. The cases where organic bodies are derived from inorganic materials by strictly chemical processes, are few, and not well marked. The chief source of these products are from the decomposition of other organic bodies under the influence of electricity, of heat, of fermentation and putrefaction, of the vital functions in the animal organism on the one hand, and by the influence of other chemical bodies on the other. Our space does not admit, else we would gladly give our readers the whole of this chapter.

The third chapter, on the Constitution of Organic Radicals, embraces many of the author's peculiar views. The following extract will show how he explains the constitution of the hydrocarbon radicals :

"By far the greater number of organic compounds are to be traced back to radicals which consist of carbon and hydrogen, and were called above *Hydro-carbyls.* By an accurate observation and comparison of these in every aspect which they present, it is found that they belong to different groups, and that those of each group mostly form an ascending series, in which each successive member contains the same number of carbon and hydrogen atoms as the preceding. But whilst the radicals of one group, in their combining proportions, are allied to hydrogen and the positive metals, those of another agree in this relation with the negative.

In all the radicals of the hydro-carbyls may be distinguished, 1. *The Active Element or Molecule;* and 2. *The Ascending Passive Component*, by which individual members of a group are formed. *In all Organic Radicals which have a positive character*, the active part *is H; in the Negative $C_2H$. The ascending passive member of the Component is always $C_2 H_2$.* All radicals which appear as simple combinations of the components $C_2 H_2$, with the active H, or $C_2H$, form the class of the *Hydroisocarbyls*, but if between the ascending $C_2 H_2$ and the active H or $C_2H$ another molecule of carbon enters $= C_2, C_4, C_6, C_8$, which may be considered as a *Nucleus*, we thus obtain the class of *Hydropolycarbyls.*"

*Hydroisocarbyls* are first treated of in the special part of the work, the first group being the Methyl group Component $C_2 H_2$ and active element H. The known members of this group are

| | | | |
|---|---|---|---|
| 1st member | Methyl | $C_2 H_2$, H | $= C_2 H_3$ |
| 2d " | Ethyl | $2 C_2 H_2$, H | $= C_4 H_5$ |
| 4th " | Valyl | $4 C_2 H_2$, H | $= C_8 H_9$ |
| 5th " | Amyl | $5 C_2 H_2$, H | $= C_{10} H_{11}$ |
| 16th " | Cethyl | $16 C_2 H_2$, H | $= C_{32} H_{33}$ |
| 24th " | Cerossyl | $24 C_2 H_2$, H | $= C_{48} H_{49}$ |
| 27th " | Cerotyl | $27 C_2 H_2$, H | $= C_{54} H_{55}$ |
| 30th " | Melissyl | $30 C_2 H_2$, H | $= C_{60} H_{61}$ |

The radicals of this group all behave towards chlorine, bromine, iodine, sulphur, selenium, etc., as hydrogen does.

The second is the formyl group, the Component being $C_2 H_2$; the active molecule formyl $= C_2H$. This is the most numerous at present known. The following members have been discovered:

| | | |
|---|---|---|
| Member | 1 Acetyl | $C_2 H_2, C_2H = C_4 H_3$ |
| " | 2 Propionyl | $2 C_2 H_2, C_2H = C_6 H_5$ |
| " | 3 Butyryl | $3 C_2 H_2, C_2H = C_8 H_7$ |
| " | 4 Valeryl | $4 C_2 H_2, C_2H = C_{10} H_9$ |
| " | 5 Capronyl | $5 C_2 H_2, C_2H = C_{12} H_{11}$ |
| " | 6 Oenanthyl | $6 C_2 H_2, C_2H = C_{14} H_{13}$ |
| " | 7 Capryl | $7 C_2 H_2, C_2H = C_{16} H_{15}$ |
| " | 8 Pelargonyl | $8 C_2 H_2, C_2H = C_{18} H_{17}$ |
| " | 9 Caprylyl | $9 C_2 H_2, C_2H = C_{20} H_{19}$ |
| " | 10 Cocyl | $10 C_2 H_2, C_2H = C_{22} H_{21}$ |
| " | 11 Laurosteryl | $11 C_2 H_2, C_2H = C_{24} H_{23}$ |
| " | 13 Myristicyl | $13 C_2 H_2, C_2H = C_{28} H_{27}$ |
| " | 14 Benyl | $14 C_2 H_2, C_2H = C_{30} H_{29}$ |
| " | 15 Palmityl | $15 C_2 H_2, C_2H = C_{32} H_{31}$ |
| " | 16 Margaryl | $16 C_2 H_2, C_2H = C_{34} H_{33}$ |
| " | 17 Stearophanyl | $17 C_2 H_2, C_2H = C_{36} H_{35}$ |
| " | 21 Behanyl | $21 C_2 H_2, C_2H = C_{44} H_{43}$ |
| " | 23 Cerossyl | $23 C_2 H_2, C_2H = C_{48} H_{47}$ |
| " | 26 Cerotyl | $26 C_2 H_2, C_2H = C_{54} H_{53}$ |
| " | 29 Melissinyl | $29 C_2 H_2, C_2H = C_{60} H_{59}$ |

The radicals of this group all possess the capability of forming acids with 3 atoms of oxygen.

These examples will suffice to explain Dr. Löwig's views of the radicals of the first division. The Hydropolycarbyls are an extremely numerous class, and are divided into five groups. Four of these are based on the proportion of the carbon nucleus, and the fifth includes volatile oils, camphors and resins.

The second division, or Carbyls, includes every radical which consists of two or more atoms of carbon, of which oxatyl= $C_2$, the radical of oxalic acid, is a type, and embrace nearly all the ordinary vegetable acids, like the oxalic, citric, malic, etc.

The third division, or Azocarbyls, includes Cyanogen and its compounds, Paraban, Fulminan and Mellan.

The fourth division, Hydroazocarbyls, includes the *uren* compounds and the organic *alkalids*, of which Glycocoll and Kreatin are members.

The fifth division, or Hydrils, embraces the organic salt bases or alkaloids, including the nitrogen bases, the paired nitrogen bases, and those containing the metals, as Kakodyl, Stibmethyl, Bismæthyl, etc.

The sixth division includes "organic combinations of a higher order."

1st. *Non-nitrogenous compounds*, as the tannic and lichen acids, coloring matters, bitter principles, common vegetable and animal matters, as celulose, starch, gum, sugar and pectin.

2d. *Nitrogenous compounds*, as the protein substances, animal tissues and coloring matters.

And lastly, the general products of decomposition, as ulmin, humin, etc.

Such is a hasty bird's-eye sketch of the "Principles" of Dr. Löwig,—too slight indeed,—yet sufficient to show how deeply he is penetrated with the importance of the Theory of Radicals, and how far he is disposed to carry it in attempting to harmonize it with the nucleus theory and other hypotheses. That many of his views of proximate constitution will melt away before the light of scientific progress there can hardly be a doubt, whilst others—and important ones too—will be confirmed and registered among demonstrable realities. The grasp of mind necessary to embrace the rapidly increasing catalogue of organic products, so as to generalize successfully, belongs to the highest order of intellect; yet in the repeated attempts that have been made to discover the laws of a natural system, theory after theory has been erected, like the scaffolding around St. Peter's, to decay away, and be renewed by each generation, until, in the progress of time, the true laws of organic combination shall have been so far established as to need them no longer.

As a book for the advanced student, the work of Dr. Löwig will prove extremely useful from its clearness and comprehensiveness, and whilst he may be disinclined to admit, or at least pause to consider many of the ultra-theoretical views of the author, as not sufficiently supported by observation and experiment, he cannot but derive the greatest interest from his clear reasoning in numerous others.

*A Clinical Phrase Book ; in English and German. Containing the usual questions and answers employed in examining and prescribing for patients ; questions in asking for and buying medicines, etc. etc.* By MONTGOMERY JOHNS, M. D. Philada. Lindsay & Blakiston ; 1853.

This unpretending little volume supplies a want often felt by those who, unacquainted with the German language, are unavoidably brought into contact with the people in Hospitals and Dispensaries, and more rarely in private practice.

The questions are arranged in the order commonly followed in examining a patient, according to the model of Recamier, given in Stillé's Elements of General Pathology ; while the answers present that latitude which will suffice to elicit the sort of information necessary to correct diagnosis.

To facilitate its use, there is attached to the volume, an English-German and German-English pronouncing lexicon, containing all the words occurring in the phrases, with the chief technical terms of medical writers and apothecaries. The convenient size of the book enables it to be readily carried in the pocket, and we feel sure that no one who knows its usefulness, as we do, will fail to possess it. Our warmest thanks are due to the author for the admirable and satisfactory manner in which he has executed his task.

